# Cadherin-Bound β-Catenin Feeds into the Wnt Pathway upon Adherens Junctions Dissociation: Evidence for an Intersection between β-Catenin Pools

**DOI:** 10.1371/journal.pone.0004580

**Published:** 2009-02-24

**Authors:** Yoonseok Kam, Vito Quaranta

**Affiliations:** Cancer Biology Department, Vanderbilt University School of Medicine, Nashville, Tennessee, United States of America; Harvard University, United States of America

## Abstract

β-catenin is an essential component of two cellular systems: cadherin-based adherens junctions (AJ) and the Wnt signaling pathway. A functional or physical connection between these β-catenin pools has been suggested in previous studies, but not conclusively demonstrated to date. To further examine this intersection, we treated A431 cell colonies with lysophosphatidic acid (LPA), which forces rapid and synchronized dissociation of AJ. A combination of immunostaining, time-lapse microscopy using photoactivatable-GFP-tagged β-catenin, and image analyses indicate that the cadherin-bound pool of β-catenin, internalized together with E-cadherin, accumulates at the perinuclear endocytic recycling compartment (ERC) upon AJ dissociation, and can be translocated into the cell nucleus upon Wnt pathway activation. These results suggest that the ERC may be a site of residence for β-catenin destined to enter the nucleus, and that dissociation of AJ may influence β-catenin levels in the ERC, effectively affecting β-catenin substrate levels available downstream for the Wnt pathway. This intersection provides a mechanism for integrating cell-cell adhesion with Wnt signaling and could be critical in developmental and cancer processes that rely on β-catenin-dependent gene expression.

## Introduction

Morphogenesis and integrity of tissues requires interactions between neighboring cells and properly coordinated regulation of gene expression [1]. In the event that these interactions are corrupt, cells may experience alterations in several core processes, including proliferation, differentiation, adhesion, and motility—all hallmarks of diseases such as cancer [2]–[4]. Interestingly, β-catenin, a multifunctional protein commonly found in excess levels in certain types of cancer [5], [6], is a key player in both calcium-dependent intercellular adhesion events and nuclear gene expression via the Wnt pathway [7]. In human malignancies, both of these functions of β-catenin are de-regulated, leading to an accumulation of protein that can cause both the loss of cell-cell adhesion and increased transcription of target genes [8].

In cell-cell adhesion, the recruitment of cytosolic β-catenin to the plasma membrane and its tight association to E-cadherin is required for formation and stabilization of adherens junctions (AJ) [9], which support proper tissue architecture and morphogenesis [10]. Cell AJ are dynamic assemblies, susceptible to both cues from their microenvironment and proper expression of their molecular components, including β-catenin [11]. For instance, when β-catenin or the catenin-binding site of E-cadherin are mutated or irregularly expressed, cell-cell adhesion is altered [12]. The fate of cadherin-bound β-catenin upon dissociation of AJ is not well understood, but it is generally agreed that it is either degraded or recycled [13].

Another pool of β-catenin, thus far thought to be separate and functionally distinct from AJ associated β-catenin [7], is an essential component of the Wnt signaling pathway, a key modulator of development that has also been implicated in cancer [6], [14]. This β-catenin pool is degraded by a pathway that involves the adenomatous polyposis coli (APC)-complex consisting of APC, axin, diversin, casein kinase I, and glycogen synthase kinase-3β (GSK-3β) [15]. Wnt pathway activation (e.g., through Frizzled receptor) leads to GSK-3β phosphorylation, which subsequently inhibits β-catenin degradation by the APC-complex [14], [16]. As a result, increased levels of cytoplasmic β-catenin accumulate and become available for nuclear translocation and binding to T-cell factor/lymphoid enhancer factor (TCF/LEF) DNA binding proteins, which change transcription of target genes [17]. This regulatory mechanism is referred to as the canonical Wnt pathway and is crucial in embryonic development across several species [14], [18]. In colorectal cancer, mutations in *APC* are found in 61% of patients [19] and result in abnormal upregulation of β-catenin-dependent transcription. Patients without *APC* mutations generally have direct alterations in β-catenin, TCF, or other molecules in the same pathway [20]–[23]. Mutation in *APC* is also the responsible initiating genetic event in the colorectal cancer syndrome familial adenomatous polyposis [5].

The functions of β-catenin are intensely studied separately in *either* AJ formation *or* Wnt signaling. However, relatively little consideration has been given to the possibility that the presence of β-catenin in *both* pathways may point to a mechanistic link between two core cell processes of fundamental importance in their own right [4], [24]. For instance, an intersection between the two β-catenin pools may serve to integrate spatial organization of cells (cell-cell adhesion) with gene expression (Wnt signaling). In a recent review, Nelson and Nusse [4] elegantly summarized the issue by asking a direct question: “Can the cadherin-bound pool of β-catenin be released and made available for signaling?” Here, we report experimental evidence that this is indeed the case.

## Results

### Perinuclear accumulation of β-catenin and E-cadherin following fast dissociation of AJ

**Figure 1:**
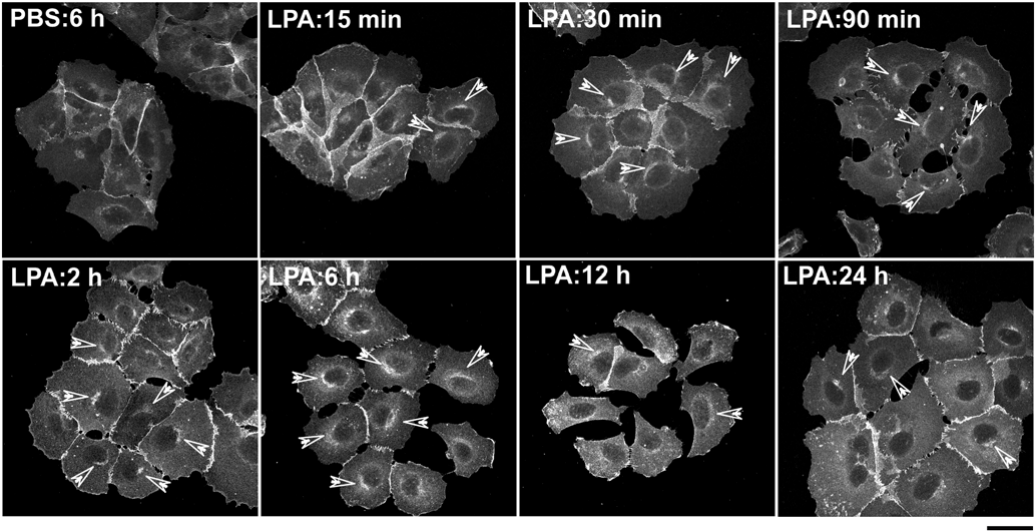
Rapid AJ dissociation by LPA induces accumulation of β-catenin in a perinuclear compartment. A431 cells were serum-starved for 18 h, incubated with PBS or 1 µM LPA for indicated times, and β-catenin expression visualized by immunofluorescence staining as described in *Materials and Methods*, using anti-β-catenin monoclonal IgG antibody followed by Alexa-Fluor 488-tagged anti-rabbit IgG antibody. Samples (N = 3 per group) were examined with a Zeiss LSM-510 confocal microscope (40× Plan-NEOFLUAR objective; NA 1.3; scale bar = 50 µm). When cells were LPA-treated, pockets of increased levels of ß-catenin accumulation (arrowheads) were visualized over time compared to PBS-treated cells; the apparent accumulation lasted up to approximately 6 h and declined as cells migrated out of colonies and rebuilt AJ formations.

**Figure 2:**
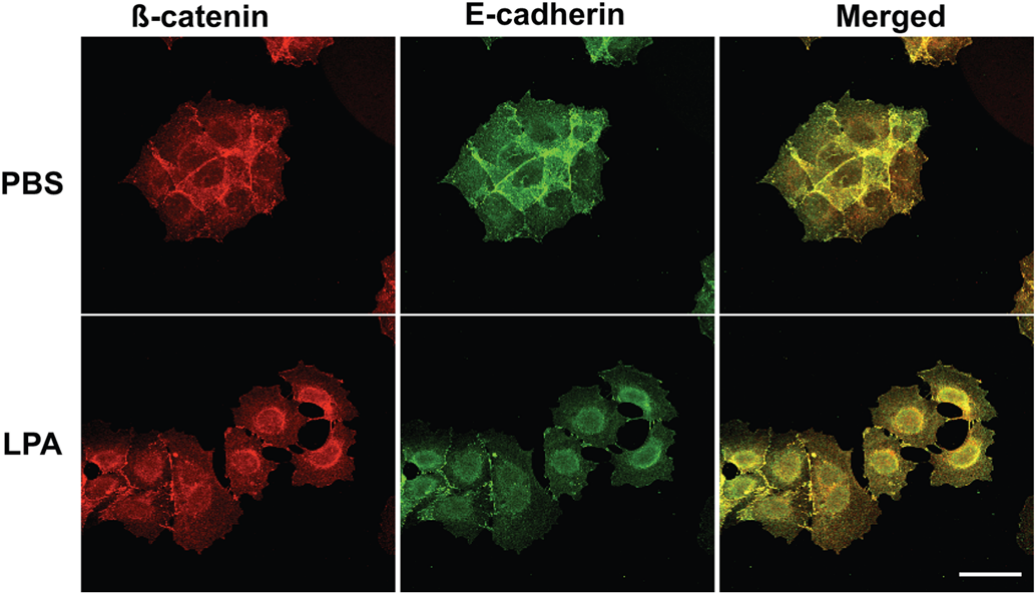
E-cadherin colocalizes with β-catenin at the perinuclear region. A431 cells were serum-starved for 18 h, pretreated with PBS or 1 µM LPA for 1.5 h, immunostained with anti-β-catenin polyclonal rabbit IgG (red) and anti-E-cadherin monoclonal mouse IgG (green), and imaged with a Zeiss LSM-510 confocal microscope (40× Plan-NEOFLUAR objective; NA 1.3; scale bar = 50 µm). When cells were LPA-treated, β-catenin expression colocalized (yellow) with E-cadherin expression. In contrast, when cells were PBS-treated for control, no colocalization was seen (N = 3 per group).

Colonies of the A431 squamous carcinoma cell line are rapidly dissociated by exposure to lysophosphatidic acid (LPA) [25]. To confirm this in our system and further focus on ß-catenin localization during this process, we pretreated A431 cells with LPA (or PBS for control) for various periods of time, immunostained cells for ß-catenin, and captured high magnification images with confocal microscopy (Figure 1). After a single application of LPA to cells at the initial time point (0 min), AJ began to disintegrate between 30–90 min, and full colony dispersal was observed between 6–12 h. However, after 12 h, cells began to reconstruct AJ formations, a task almost completed after 24 h. In dissociating cells, dense β-catenin immunostaining can be observed in a perinuclear compartment residing at one side of the nucleus (Figure 1; arrows). This apparent accumulation of internalized β-catenin in the perinuclear compartment lasted up to 6 h, and declined as cells migrated out of colonies (12 h) or rebuilt AJ (24 h). In contrast, PBS-treated cell colonies showed no change in AJ formation, nor accumulation of β-catenin at any time (6 h only shown in Figure 1). In addition to β-catenin, AJ components E-cadherin and α-catenin were also disorganized by LPA treatment, as expected in disintegrating AJ (Figure S1). Since E-cadherin is tightly bound to β-catenin at AJ, we also examined LPA-treated (and PBS-treated control) A431 cells for colocalization of these molecules (Figure 2). When cells were pretreated with LPA, they displayed perinuclear accumulation of E-cadherin (green) along with β-catenin (red), consistent with co-trafficking to the same compartment (colocalization in yellow in merged image) (Figure 2). In contrast, PBS-treated cells displayed no perinuclear accumulation of either marker.

### Translocation of β-catenin from AJ to the endocytic recycling compartment

**Figure 3:**
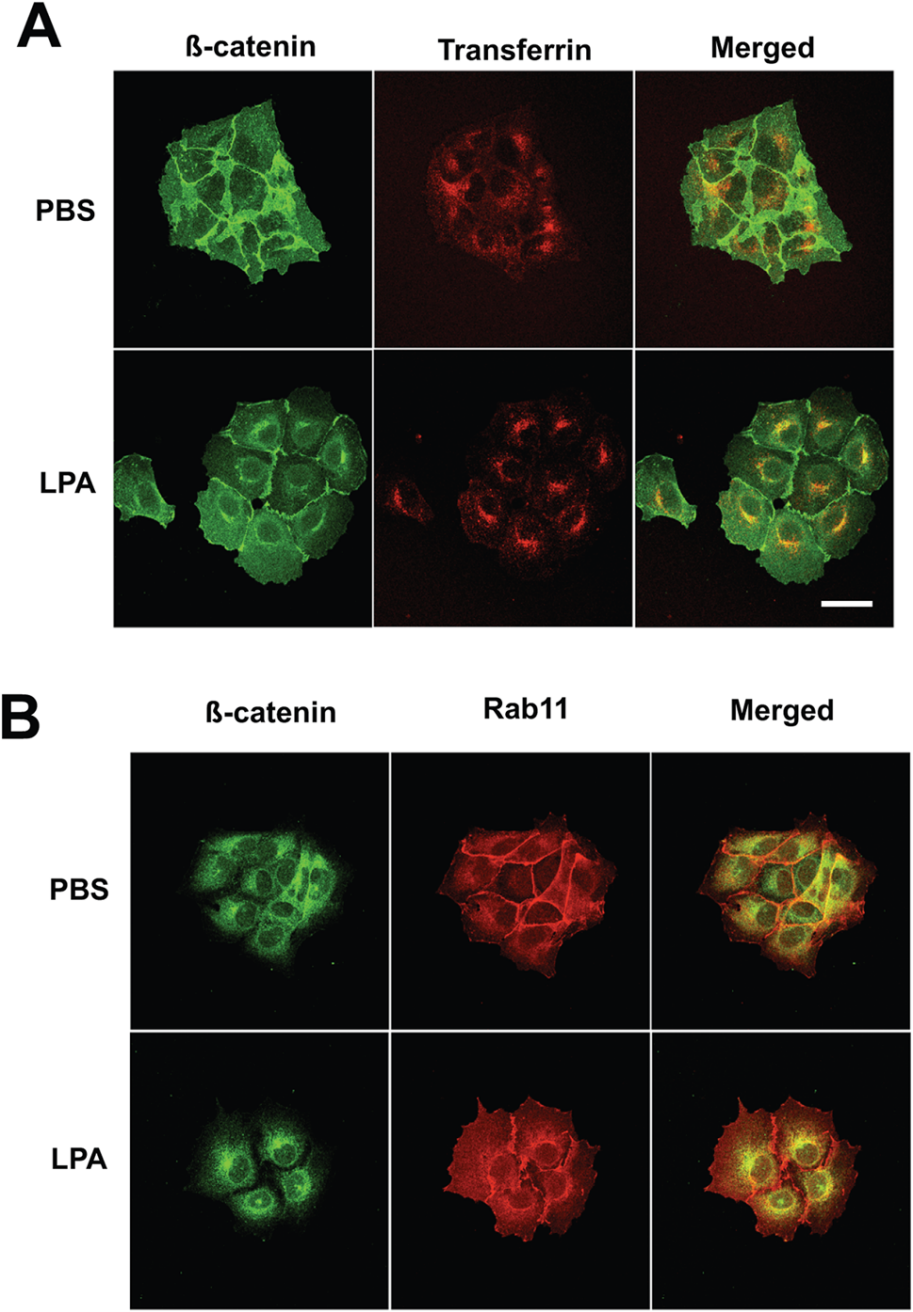
β-catenin is targeted to the perinuclear endocytic recycling compartment. (A) A431 cells were serum-starved, pretreated with 1 µM LPA for 1 h, and incubated for 30 min in 10 µg/ml Alexa-Fluor™ 647-transferrin (colored as green). Cells were fixed, stained with anti-β-catenin monoclonal IgG antibody (colored as red), and imaged using a Zeiss LSM-510 confocal microscope (40× Plan-NEOFLUAR objective; NA 1.3; scale bar = 50 µm). The perinuclear region stained positively for transferrin under receptor recycling conditions and overlapped well, colocalizing with ß-catenin staining. (B) The distribution of β-catenin at the perinuclear region was also performed with anti-Rab11 polyclonal rabbit antibody. Cells were incubated in PBS or 1 µM LPA for 1.5 h, fixed, stained with anti-β-catenin monoclonal antibody (red) and Rab11 antibody (green), and imaged with a Zeiss LSM-510 confocal microscope. Rab11 staining was also found to highly colocalized with β-catenin staining (yellow; arrows) (N = 3 per group).

In order to positively identify the specific perinuclear compartment(s) where β-catenin and E-cadherin accumulated, we also compared localization of β-catenin with transferrin. Transferrin bound to its cell surface receptor is internalized by endocytosis, and has been well characterized as a classic marker for the perinuclear endocytic recycling compartment (ERC) [26]. As shown in Figure 3A, LPA-treated cells displayed accumulation of both β-catenin (red) and transferrin (green) under receptor recycling conditions. These stains colocalized in particular regions of the cell near the nucleus (yellow regions of merged images; arrows). In contrast, colocalization of β-catenin and transferrin is not observed in PBS-treated cells, although both markers stained loosely throughout these cells. We further compared distribution of β-catenin in LPA-treated cells with that of markers for other intracellular vesicular structures, including Rab11 (ERC), LAMP-1 (lysosomes), Golgin-97 (Golgi), and EEA1 (early endosomes). Of these markers, only Rab11 (Figure 3B; green) and EEA1 (Figure S2) were found to colocalize with β-catenin (red), with Rab11 exhibiting almost perfect colocalization (yellow areas of merged images, indicated by arrows), and EEA1 to a lesser extent. In contrast, PBS-treated cells stained for all markers, but did not display any patterns of colocalization (Figure 3; Figure S2). These results indicate that, upon rapid dissociation of AJ (induced by LPA), β-catenin (presumably still complexed with E-cadherin) is internalized and transported to the perinuclear ERC.

### Translocation of AJ associated β-catenin from the ERC to the nucleus

**Figure 4:**
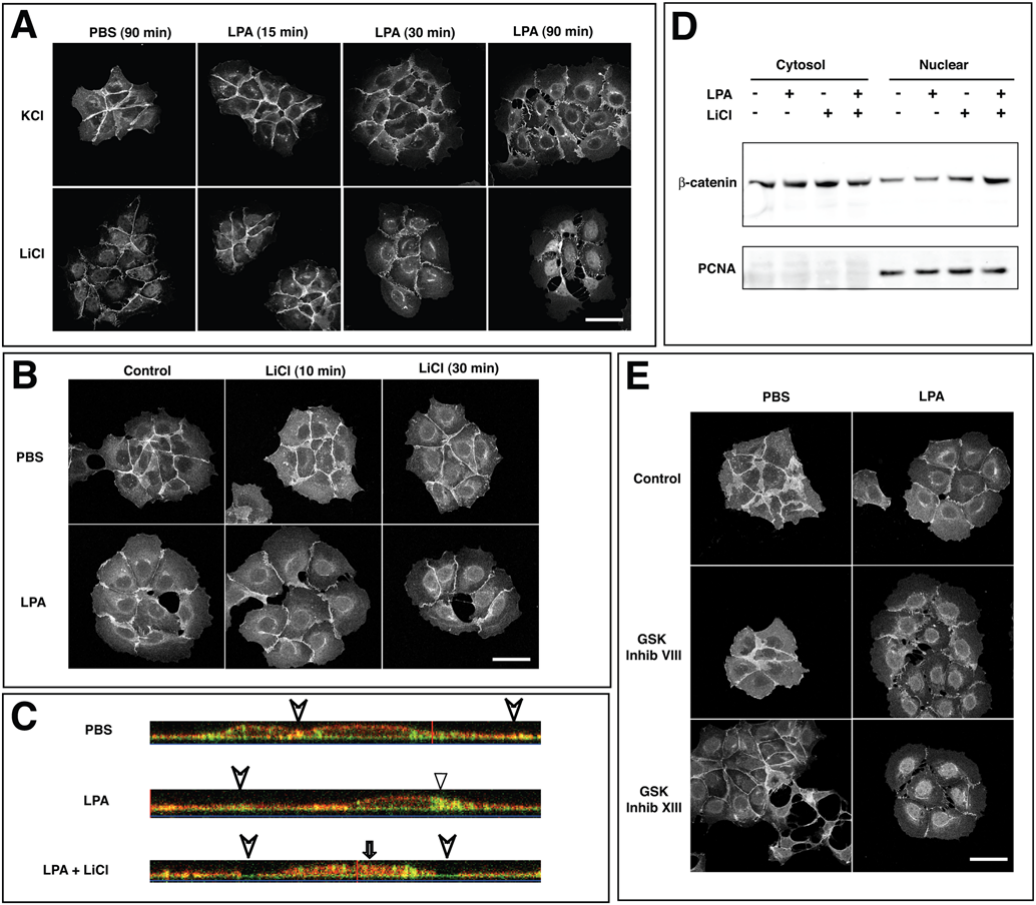
ERC targeted β-catenin translocates to the nucleus in the presence of Wnt activator or GSK-3β inhibitors. (A) A431 cells were serum starved for 18 h, incubated in PBS or 1 µM LPA for indicated time, then treated with either 40 mM KCl or LiCl for 5 min, immunostained for β-catenin using a monoclonal IgG antibody, and imaged with a Zeiss LSM-510 confocal microscope (40× Plan-NEOFLUAR objective; NA 1.3; scale bar = 50 µm). Cells treated with both LPA and LiCl displayed concentrated β-catenin accumulation in their nuclei (especially at 90 min when AJ dissociation occurs); whereas, those cells pretreated with PBS and KCl/LiCl together or LPA and KCl together (control salt) displayed no nuclear accumulation. (B) Cells were serum starved, treated with PBS or 1 µM LPA for 1.5 h, subsequently incubated in the presence or absence of 40 mM LiCl for indicated time, immunostained for β-catenin, and imaged. LPA-treated cells accumulated β-catenin at the ERC and showed higher catenin nuclear translocation than control upon treatment with LiCl, particularly at the 30 min time point. (C) Cells were serum-starved, stimulated with LPA or PBS for 1 h, pretreated with LiCl as indicated, and further incubated with an antibody against β-catenin and Alexafluor 647-transferrin for 30 min. The lateral images of cells were reconstituted from confocal z-sectioned images (β-catenin, red; transferrin, green). This view captures β-catenin co-distribution with transferrin in the perinuclear ERC when cells were stimulated with LPA alone (arrow head). Cells treated with both LPA and LiCl displayed accumulation in the central nuclear region (open arrow). In both cases, AJ β-catenin levels (arrows) were reduced. (D) Serum-starved cells were stimulated with PBS or LPA±LiCl as indicated, and the nuclear and cytoplasmic fractions were isolated. Western blot using anti-β-catenin (top panel) and anti-PCNA monoclonal antibodies (lower panel) was performed according to the Odyssey Infrared Imaging System. LiCl-LPA costimulation increased β-catenin level normalized by PCNA band intensity, compared to LPA or LiCl alone (N = 3). (E) Cells were stimulated by PBS or LPA for 1.5 h, incubated in the presence or absence of 1 µM GSK-3β inhibitor VIII or XIII for 30 min, and immunostained for β-catenin. Addition of specific GSK inhibitors resulted in β-catenin translocation to the nucleus in LPA-treated, but not control cells (N = 3).

In addition to its role as an AJ component, β-catenin also plays an important role in the nucleus as an activator of the transcription factor TCF/LEF [17]. Wnt-stimulation and *APC* mutations are known to inactivate GSK-3β and result in β-catenin translocation from the cytoplasm to the nucleus. To test whether ERC-targeted β-catenin from disrupted AJ could be translocated to the nucleus upon Wnt activation, we exposed LPA-treated cells to lithium chloride (LiCl), a GSK-3β inhibitor often used for stimulating downstream activation of the Wnt pathway [27], [28]. In PBS-treated cells, no nuclear accumulation of β-catenin was observed upon treatment with either LiCl or potassium chloride (KCl; a control salt) (90 min shown only; Figure 4A). Likewise, no nuclear accumulation of β-catenin occurred in cells treated with LPA and control salt, KCl. In contrast, when cells were pretreated with both LPA (for AJ disassociation) and LiCl (for Wnt pathway activation), β-catenin accumulated in the nucleus (Figure 4A), at the time point (90 min) when AJ dissociation begins (see Figure 1). This result supports the possibility that AJ-bound β-catenin can be fed into the Wnt pathway after AJ dissociation. Several additional experimental approaches were used to confirm this possibility, as described below.

It has been reported that extended exposure (several days) of cells to LiCl (generally>10 mM) may have toxic effects [29]–[31]. In our experiments, we used 40 mM LiCl, however only incubated cells for 1–3 h, depending on experimental setup. However, to completely diminish this possibility from having any effect on our results, we stimulated A431 cells with LPA or PBS for 90 min and then pulsed cells with LiCl for only 10 or 30 min, followed by immunostaining for β-catenin. As shown in Figure 4B, these shorter treatments with LiCl still caused nuclear translocation of ERC-resident β-catenin in LPA-treated cells, more so at 30 min than 10 min. These results further support the idea that Wnt activation acts on AJ dissociated β-catenin.

To confirm that, in these experiments, ERC-resident β-catenin was translocated inside the nucleus, we reconstructed its physical location by confocal microscopy generated z-stack analyses. Figure 4C shows a lateral view of an immunostained representative cell captured from each treatment (PBS, LPA, or LPA+LiCl). Upon LPA treatment alone, cells exhibited β-catenin expression (red) colocalized with transferrin (green) in the perinuclear ERC (arrow head), compared to PBS control. However, when both LiCl and LPA were added, colocalization instead occurred in the central nuclear region of cells (solid arrow). AJ β-catenin expression (arrow) is obviously reduced in both cases. These results confirm that AJ associated β-catenin was translocated into the nucleus given co-stimulation with LPA (for dissociation of AJ) and LiCl (for GSK-3ß inhibition).

To further confirm the microscopy results with an independent approach, we lysed cells and performed cytosol/nuclear fractionation followed by western blotting with antibodies specific for β-catenin or PCNA, a nuclear antigen. As shown in Figure 4D, co-stimulation with LPA and LiCl increased the β-catenin level in the nuclear fraction (normalized by PCNA band intensity) by 2.4 fold (compared to−/−band), while LPA or LiCl alone induced almost no change or a 1.4 fold-increase, respectively. These results align with our previous findings obtained from microscopic analysis.

It has been suggested that GSK-3β inhibition by LiCl releases β-catenin from its fate of proteasome-mediated destruction [27], [28], [32]. Since LiCl may have additional effects on cells beside GSK-3β inhibition, we also tested more specific reagents, GSK inhibitor VIII and XIII, which inhibit GSK-3β. As seen in Figure 4E, addition of these specific GSK inhibitors also resulted in β-catenin translocation to the nucleus in LPA-treated, but not in PBS-treated control cells. These results further strengthen our previous findings that given GSK-3β inhibition (via LiCl treatment), β-catenin accumulation translocates to the nucleus of the cell following dissociation of AJ.

### Pulse-chasing of β-catenin from AJ to the nucleus

**Figure 5:**
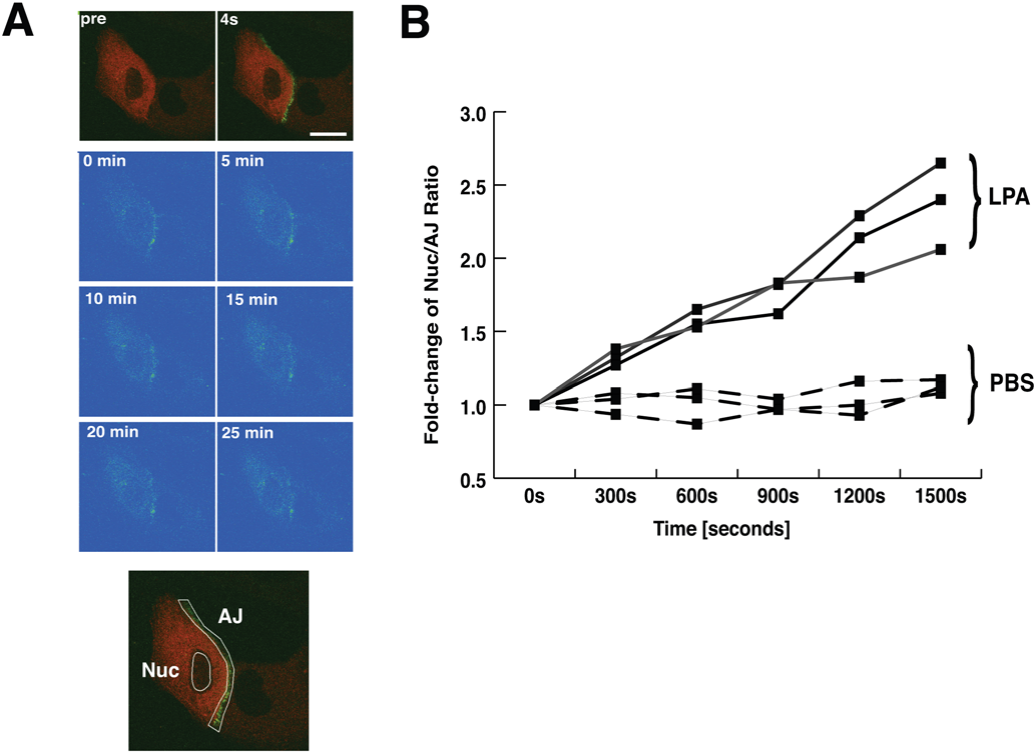
β-catenin from the cadherin-bound AJ pool translocates to the nucleus. (A) β-catenin-ΔGSK-PA-GFP construct (green) was co-expressed in A431 cells with mRFP-actin (red). The AJ area was photo-activated selectively by 405 nm light using a Zeiss LSM510 confocal microscope (pre, pre-activation; 4 s, 4 sec after activation). The fluorescence of PA-GFP (green) was monitored at the indicated times, together with of mRFP-actin (red). One µM LPA stimulation images are shown in panel (PBS-treated cells not shown). The fluorescence levels of the nuclear (Nuc) and AJ areas (representative areas shown in bottom image) were measured over time using Zeiss LSM510 software. (B) LPA-stimulated cells exhibited a steady decrease of AJ expression, coupled with a steady increase of nuclear β-catenin expression, over time. In contrast, PBS-treated cells showed a steady level of nuclear β-catenin. Since cells displayed a wide variety of morphologies and initial fluorescence intensities per region, all measurements were normalized by plotting data as the fold-change of the fluorescence ratio (against corresponding measure for 0 h) of nuclear fluorescence/AJ fluorescence over time, for both PBS- and LPA-stimulated cells (N = 3). LPA-treated cells displayed significantly (P<0.05) increased levels of nuclear β-catenin over time, compared to PBS-treated control cells.

Our data to this point clearly indicate that rapid disruption of AJ results in increased nuclear β-catenin when GSK-3β function is impaired. However, microscopic images including immunofluorescence staining or western blot data present only a stationary snapshot of β-catenin localization and do not provide information about dynamic translocation of β-catenin. In order to obtain direct evidence that the β-catenin that is subsequently localized in the nucleus truly originated from the AJ pool, we monitored β-catenin movement in living cells by pulse-chasing β-catenin fused with photoactivatable (PA)-GFP [33]. Since exogenous wild type β-catenin is subject to GSK-3β-mediated destruction, and is not easily incorporated into AJ (A. I. Barth, Stanford University, personal communication), we used a β-catenin construct that cannot be phosphorylated by GSK-3β due to point mutations at four important phosphorylation sites [34]. We subcloned β-catenin into the PA-GFP expression vector and produced β-catenin-ΔGSK-PA-GFP (β-catenin-Δ-GFP). As shown in Figure 5A, β-catenin-Δ-GFP was photoactivated (top row of images labeled pre and 4s) with a 405-nm laser beam specifically at a narrow area around AJ, and fluorescence intensity of both AJ and nuclear regions of cells were measured at various time points over 25 min (LPA-stimulated cell shown; PBS-treated images not shown). PBS-treated cells displayed relatively stable expression of nuclear β-catenin, and a steady decline in AJ localized β-catenin. In contrast, when AJ were dissociating in LPA-treated cells, β-catenin expression at the AJ region rapidly decreased, while nuclear β-catenin fluorescence levels increased continuously. Since cells displayed a wide range of morphologies and initial fluorescence intensities per region, we normalized measurements to reflect the ratio of nuclear fluorescence to AJ fluorescence per cell, versus the original fluorescence measurements for that particular cell as described in *Materials and Methods* (Figure 5B). Figure 5B summarizes the fold-change of the fluorescence nuclear/AJ ratio measured over time, for both LPA- treated (solid lines) and PBS-treated (dotted lines) cells. LPA-treated cells exhibited significantly (N = 3; P<0.05) higher nuclear/AJ ratios than PBS-treated cells, and the decrease of AJ associated β-catenin paired well with the increase of nuclear expression. The nuclear increase in fluorescence level in this assay can only be a result of β-catenin molecular translocation from the junctional regions, since only β-catenin-ΔGSK-PA-GFP emits any degree of fluorescence. We submit that this latter result is direct evidence that cadherin-bound β-catenin, originating from dissociating AJ, travels to a site, the ERC, where it becomes a substrate for Wnt signaling. Conjugation with PA-GFP allowed us to directly tag β-catenin at the site of AJ and follow its fate upon AJ dissociation, without interference from new protein synthesis.

### TOPflash transcriptional analysis of A431 and HEK293 cells

**Figure 6:**
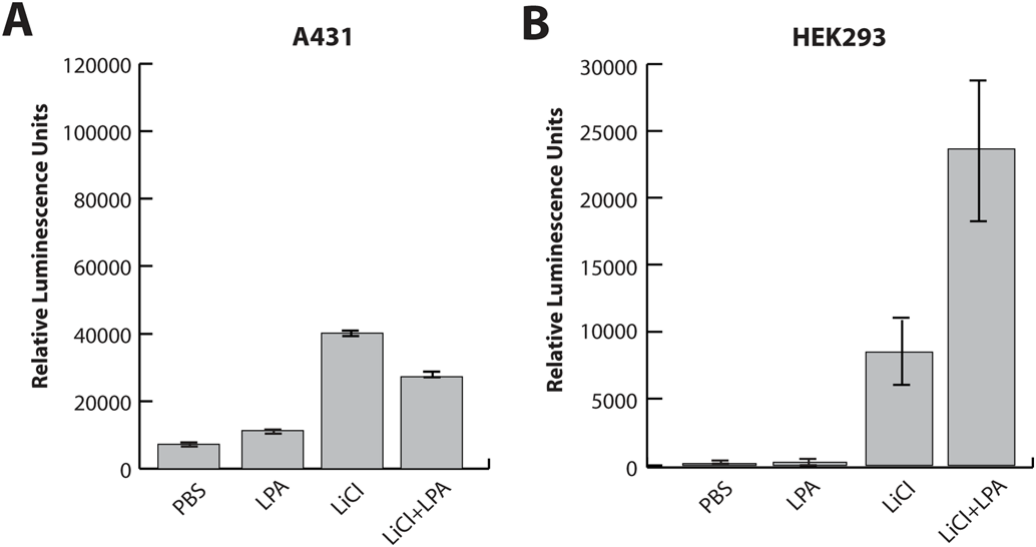
TCF/LEF-dependent transcriptional activity TOPflash. In order to measure TCF/LEF-dependent transcriptional activity of A431 and HEK293 cell lines, cells were transfected with a TOPflash reporter plasmid. After overnight starvation, cells were stimulated by PBS or LPA, in the presence or absence of LiCl for 18 h, and were assessed for transcriptional activity using the Steady-Glo Luciferase assay system. (A) A431 cells treated with either PBS or LPA alone showed negligible levels of transcriptional activity (N = 3). In contrast, cells treated with LiCl alone showed significant increase in signal (N = 3; P<0.0001), which was significantly diminished by co-treatment with LPA (N = 3; P = 0.001). (B) Similarly, HEK293 cells treated with either PBS or LPA alone showed negligible levels of transcriptional activity (N = 3). Cells treated with LiCl alone showed significant increase in signal (N = 3; P = 0.024), which was significantly diminished by co-treatment with LPA (N = 3; P = 0.017).

In order to see the effect of LPA-enhanced nuclear translocation of β-catenin on gene expression, we measured TCF/LEF-dependent transcriptional activity using a TOPflash reporter plasmid. After overnight starvation, cells were stimulated by PBS or LPA, in the presence or absence of LiCl for 18 h, and assessed for transcriptional activity by measuring luciferase activity. A431 cells showed significantly elevated LEF/TCF dependent transcription with LiCl alone (compared to PBS control; N = 3; P<0.0001; Figure 6A). In contrast, little change occurred with LPA treatment alone and, surprisingly, the LiCl-induced transcriptional elevation was significantly diminished by co-treatment with LPA (N = 3; P = 0.001).

We also tested several additional cell lines, with or without mutation in APC or beta-catenin, and found that the effect of LPA on canonical Wnt-induced transcription varies depending on cell line. For example, LiCl significantly increased transcription levels of HEK293 cells (Figure 6B; N = 3; P = 0.024), a system well-known to be sensitive to canonical Wnt3a signaling activation (35); however, in direct contrast to A431, co-treatment with LiCl and LPA showed a rather synergistic effect on transcription of HEK293 cells (Figure 6B). Interestingly, LPA induced both A431 and HEK293 cell scattering (unpublished results).

## Discussion

**Figure 7:**
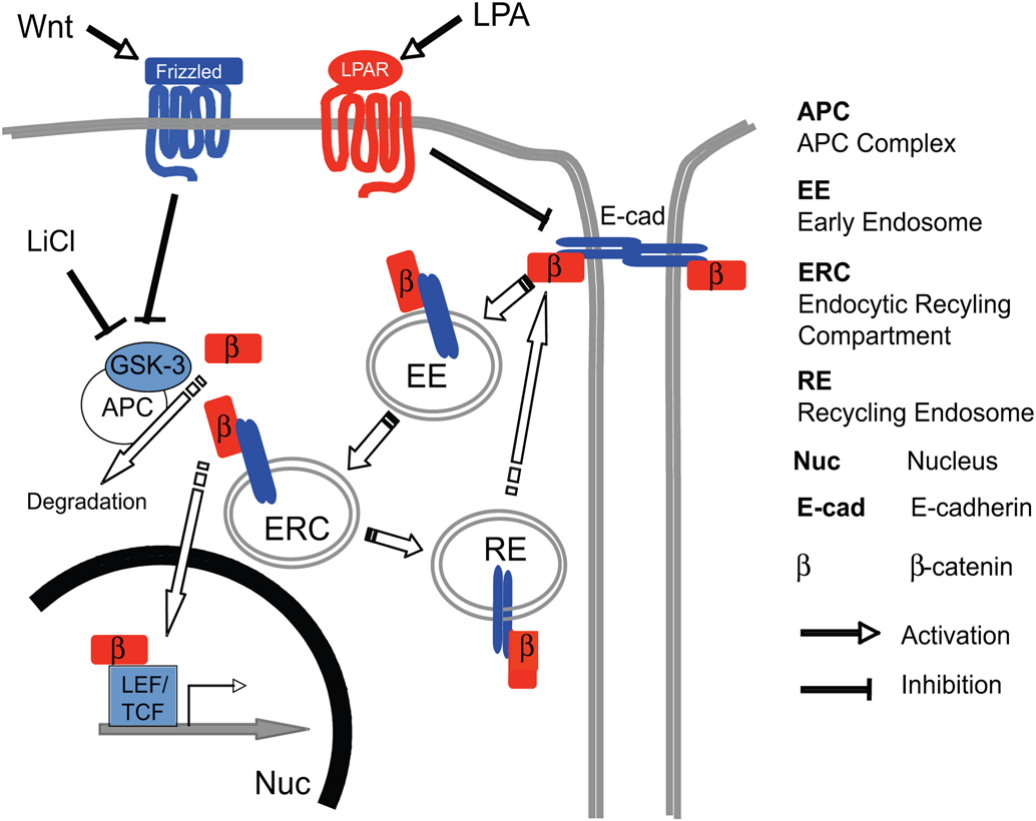
A schematic model for the intersection of cadherin-bound β-catenin pool with Wnt-signaling upon AJ dissociation by LPA stimulation. ß-catenin (ß) is recruited to sites of cell-cell interactions and binds E-cadherin (E-cad) establishing adhesion via formation of adherens junctions (AJ). Upon LPA stimulation, AJ dissociate and cadherin-bound ß-catenin travels to the early endosome (EE), endocytic recycling compartment (ERC) [and potentially the recycling endosome (RE) before its exit back to AJ area], before being translocated into the nucleus (Nuc) of the cell, where it engages transcription factors such as TCF and LEF (altering gene expression). Translocation is dependent upon LiCl treatment or Wnt pathway activation (via binding to receptors encoded by frizzled genes), as these steps prevent glycogen synthase kinase 3ß (GSK3ß) from phosphorylating the (APC) complex and ß-catenin substrate (ß). Unphosphorylated ß either degrades or escapes to the nucleus where it engages transcription factors such as TCF and LEF.

We present evidence that β-catenin from dissociating AJ can reach a perinuclear location, the ERC, from which it can be translocated into the nucleus upon activation of the Wnt pathway. In our experiments, we took advantage of the ability of LPA to cause rapid and synchronous dissociation of AJ in epithelial cell colonies [25], presumably enhancing levels of β-catenin along this intracellular route and facilitating its detection. Thus, in LPA-treated A431 cell colonies we found that: 1) β-catenin and E-cadherin are colocalized by immunostaining in a discrete region near the nucleus; 2) this region is identical to the perinuclear ERC, as determined by co-staining with transferrin (under receptor-recycling conditions) and a classic ERC marker, Rab11, but not by markers for others parts of the endocytic pathway; 3) ERC-resident β-catenin translocates to the nucleus upon treatment with a Wnt pathway activator, LiCl, or with specific inhibitors of GSK-3β, which is known to phosphorylate β-catenin and promote its degradation; and 4) perhaps the strongest direct evidence that AJ-bound β-catenin feeds into the Wnt pathway was obtained by experiments in which AJ-bound β-catenin, fused with PA-GFP, was selectively fluorescent tagged by laser activation, and then followed by quantitative microscopy as it accumulated into the nucleus. Based on our set of results, we propose the existence of a pathway that physically feeds AJ β-catenin to Wnt signaling (Figure 7).

Coordination between cadherin-bound and Wnt-signaling pools of β-catenin has been addressed previously, both in theoretical and experimental form. Nelson and Nusse (2004) [4] provided a conceptual framework for intersection of these β-catenin pools in the context of differentiation and development. Gottardi and Gumbiner [7] showed in elegant experiments that folding distinguishes nuclear from cytoplasmic β-catenin, and proposed this difference may determine the binding partner and thus the functional choice of β-catenin. Additionally, Brembeck *et al.* [36] found they could bias intracellular distribution of β-catenin by overexpressing the β-catenin binding protein BCL9-2 in the nucleus. These experimental reports are generally centered around the idea that the balance between AJ and nuclear β-catenin is regulated biochemically, and that adhesion and transcription functions of β-catenin are relatively independent and coordinated by differential allotment of available β-catenin to the two functionally distinct pools.

In contrast, our present study points to an additional, more direct mechanism of coordination that links adhesion and transcription function of β-catenin in a more straight-forward manner, which suggests functional integration between adhesion and Wnt-dependent differentiation. Of course, since multiple mechanisms may control pool distribution of β-catenin, studies are warranted to determine how these mechanisms may interface with each other. Thus, the AJ-Wnt β-catenin transport pathway we describe here does not conflict with, but rather complements, biochemical regulation of β-catenin allotment to distinct pools.

A legitimate question is why an AJ-to-Wnt connection for β-catenin via the endocytic pathway has not been recognized before, given the intense scrutiny both AJ and Wnt signaling have been under in numerous laboratories. We hypothesize that rapid and synchronous dissociation of AJ by LPA is a key to our observation, because it releases sufficient amounts of cadherin-bound β-catenin to overload the ERC compartment and allows for detection by current microscopy techniques. Images similar to our own were included in a report by Ivanov *et al.* [37], whereby AJ were rapidly dissociated by Ca^++^ removal. We repeated those experiments and obtained results comparable to LPA treatment, although ERC localization and nuclear translocation of β-catenin were partially confounded by side effects on cell morphology, possibly induced by the Ca^++^ deprivation (Figure S3). Nonetheless, the Ca^++^ deprivation result further supports the idea that large amounts of β-catenin, suddenly released from AJ, are key to detecting its intracellular route to the ERC and nucleus.

LPA is a versatile bioactive serum molecule [38], [39] that binds to specific G-protein coupled receptors and is involved in many physiological processes including cell survival, proliferation, differentiation, and motility [40], [41], as well as cancer progression [42]. We recently reported dispersal of cancer cell colonies by LPA [25], followed by morphological changes resembling epithelial mesenchymal transition [43], a process associated with β-catenin-mediated transcriptional activity [44]. Furthermore, LPA receptors have been shown to increase cell proliferation in *APC*-mutated colorectal cancer cells by inhibiting β-catenin degradation via Wnt signaling modulation lines [45]. However, the role of disruption of AJ was not investigated and the connections between LPA-induced morphological changes and the Wnt pathway remain to be investigated.

Future studies will also have to address how and where the ERC-accumulated β-catenin interacts with the APC complex and GSK-3β. Preliminary experiments performed in our laboratory indicate that a proteasome inhibitor MG132, when added to LPA-treated cells, also promotes translocation to the nucleus (Figure S4). This result implies either the existence of an ERC-to-proteasome pathway or coupling between the two that leads to slow degradation of excess β-catenin internalized from dissociating AJ. Palmer *et al.* [46] performed fractionation experiments and found that proteasome molecules were present in cytosolic, nuclear, and microsomal fractions (primarily the ER and cis-Golgi); however it is theoretically possible that proteasomes could become associated with the ERC, since ERC-accumulated proteins are subjected to be recycled to plasma membrane through trans-Golgi in a Rab11-dependent manner [47]. Furthermore, it suggests that the capacity of this ERC-based regulation of β-catenin degradation may be exceeded under some conditions, for example when large amounts of β-catenin “spill over” or the proteasome is impaired.

It is presently unclear which endocytic mechanism is employed for internalization of cadherin-bound β-catenin. It is perhaps natural to expect that β-catenin is internalized together with E-cadherin, since they are colocalized at the ERC region (Figure 2), and β-catenin presumably faces the cytoplasm for further interaction with other molecules. E-cadherin internalization and intracellular trafficking have been studied intensively [13]. Ivanov *et al.* [37] suggested that E-cadherin and β-catenin are endocytosed by clathrin-dependent mechanisms and localized in early endosomes. However, others have suggested that E-cadherin internalization is mediated by clathrin-independent endocytosis [13]. Lu *et al.* [48] proposed that EGF-induced AJ down-regulation is mediated via caveolae. More recently, EGF-induced internalization of E-cadherin-catenin complex was suggested to be mediated by Rac-modulated macropinocytosis [49]. Since ERC is a common compartment used for recycling after endocytosis and has a longer lifetime than other vesicular structures, these reports, though not conclusive, are consistent with β-catenin reaching the ERC, where it may intersect Wnt signaling.

Interestingly, clathrin-dependent endocytic internalization of Wnt protein itself was recently shown to be required for full activation [50]. The internalized Wnt ligand was targeted to the same region where internalized transferrin accumulated–presumably the ERC. Taken together with our results, these observations raise the possibility that ERC may be an intracellular site where Wnt-signaling molecules and internalized AJ β-catenin intersect. In addition, two recent reports showed a requirement of GSK-3ß for endocytic recycling of integrin [51] and Glut1 [52]. Our preliminary result from immunofluorescence staining of GSK-3β, a target of Wnt signaling, also showed that it overlapped well with transferrin and β-catenin in cells stimulated by LPA (unpublished data). While these data further support intersection of AJ β-catenin with Wnt pathway at the ERC, different vesicular compartments either upstream or downstream of ERC should also be considered. Thus, while the characterization of intracellular transport mechanisms that support the journey of β-catenin from AJ to ERC are outside the scope of this manuscript, it poses some intriguing challenges and should stimulate more detailed studies in the future.

On a speculative note, the AJ-to-Wnt β-catenin connection provides an intuitive mechanism to integrate cell-cell adhesion with gene expression (i.e., spatial tissue organization with cell differentiation). More explicitly, the implications of an intersection between cell-cell adhesion and Wnt pathways are manifold. First, in this scenario, AJ integrity becomes a key factor to determine susceptibility to Wnt signaling. For instance, at constant Wnt activity, the amount of β-catenin that reaches the nucleus may increase if AJ are disrupted. That is, the intensity of Wnt signaling response could be affected by AJ stability. Thus, cells would modulate gene expression by integrating AJ status with Wnt-like external signaling. Second, the effects of the AJ/Wnt axis may be compounded by other transcriptional changes induced by AJ disruption. For example, a recent report [53] suggests that, after fibroblast growth factor (FGF) stimulation, FGF receptor is translocated to the nucleus in an E-cadherin-dependent manner. In pathological contexts, AJ disruption may have abnormal, constitutive effects in cell lines that carry mutations in the Wnt pathway. For example, we have found that several colorectal cancer cell lines with *APC* mutations show more nuclear accumulation of β-catenin when treated with LPA (unpublished data), which is consistent with previous reports [45], [54]. Therefore, it appears that *APC* mutations uncouple AJ stability from Wnt-mediated regulation and, whether or not the Wnt pathway is activated, cadherin-bound β-catenin traveling from dissociated AJ may end up in the nucleus and presumably alters transcriptional regulation.

While our results clearly demonstrate that the cadherin-bound pool of β-catenin can be made available to Wnt signaling [4], it has been difficult, in our hands, to directly prove physiological significance of this intersection experimentally. This difficulty stems in large part from our observation that the increase of AJ β-catenin in ERC, and subsequently the nucleus upon Wnt activation (via LiCl+LPA), does not translate directly into activation, or super-activation of canonical Wnt target genes in A431 cells (Figure 6A). Further, this antagonistic effect was even more pronounced in Wnt3a/LPA co-treated cells (Figure S5). On a side note, while Wnt3a is the physiological relevant ligand, we focused on the steps related to β-catenin degradation, following accumulation in the ERC. LiCl appears to be a better reagent for this, since it simply prevents β-catenin degradation by inhibition of GSK3-β, presumably without the additional effects that Wnt3a may have. Therefore, the majority of experiments were carried out with LiCl stimulation.

The β-catenin-ΔGSK-GFP mutant used in photo-activation experiments also revealed that LPA suppresses the gene expression that is elevated by mutating β-catenin (Figure S6). Results from several additional cell lines, with or without mutation in APC or beta-catenin, further revealed that the effect of LPA on Wnt-induced transcription varies depending on cell line. For example, no clear pattern could be discerned among various colorectal cancer cell lines (HCT-8, HCT-15, HCT-116, HT-29) we tested (Figure S7). However, LPA showed a rather synergistic effect on the transcription with LiCl in HEK293 cells (Figure 6B).

There are multiple possible explanations for these mixed results. The first possibility is that the cadherin-bound/Wnt-bound β-catenin intersection is devoid of physiological significance. The second possibility is that regulation of Wnt-dependent gene expression is more complex than we expected, and increased levels of AJ-bound β-catenin are compensated for by other mechanisms (e.g., non-canonical Wnt pathway suppression of canonical Wnt activation [55]). A third possibility is that LPA acts like non-canonical Wnt signals (e.g., Wnt5) that repress canonical Wnt signaling. There was a previous report that Wnt5 suppresses LEF/TCF dependent gene expression, without changing nuclear translocation of β-catenin (56). Interestingly, LPA downstream signaling networks share common nodes with non-canonical Wnt signaling, such as calcium, though there are currently no reports of these being antagonists of canonical Wnt. Another possibility is that APC mutations, or lack thereof, could make a significant difference. It has been reported that even with nuclear β-catenin elevated, transcription of target genes is still under tight regulation by APC (57). This hypothesis is supported by our data showing the antagonistic effect of LPA on the LEF/TCF-dependent transcription induced by mutant form of β-catenin, β-catenin-ΔGSK-GFP (Figure S6). In addition, a recent report suggested that β-catenin is also subjected by further regulation at the plasma membrane to be ‘competent transcriptionally’ in APC and Wnt-receptor complex dependent manner (58). Yet another possibility is that fast recycling of β-catenin to re-form AJ might be induced by LPA. LPA increases lamellipodia formation in A431 cells. This event is expected to be related with Rho and Rac small G-protein activities, which are tightly involved in the initiation and expansion of AJ (59). If LPA can promote new AJ formation, the fast consumption of β-catenin for AJ from the ERC can be considered as a possible mechanism for LPA-induced downregulation of Wnt signaling, vis-à-vis increased nuclear concentration.

In a sense, it is not surprising that gene expression, in differentiated cells, would be “protected” by several alternative fail-safe mechanisms. Future experiments, including some planned in our laboratory, will have to address the issue of how to unveil the physiological significance of this novel link between AJ- and Wnt-associated β-catenin.

## Materials and Methods

### Materials

L-α-lysophosphatidic acid (oleoyl, LPA 18∶1) was purchased from Avanti Polar Lipids (Alabaster, AL), and stored at −80°C at a stock concentration of 100 µM. The monoclonal antibodies against β-catenin, E-cadherin, PCNA, and EEA1 were obtained from BD Biosciences (San Jose, CA). The polyclonal rabbit IgG antibody against β-catenin, the mAb rabbit IgG against Rab11, mouse IgG mAb against Golign97, Alexa-Fluor™488-anti-rabbit IgG, Alexa-Fluor™568-anti-mouse IgG, and Alexa-Fluor™647-transferrin were purchased from Invitrogen (Carlsbad, CA). GSK inhibitor VIII and XIII, and proteasome inhibitor MG132 were purchased from EMD Biosciences (Gibbstown, NJ). KCl and LiCl were purchased from Sigma (St. Louis, MO). The construct expressing β-catenin-ΔGSK-GFP was a generous gift from A. I. Barth and W. J. Nelson, (Stanford University) and the vector containing photo-activatable-(PA)-GFP-C1 cDNA was a gift from A. Kenworthy (Vanderbilt University). The expressing plasmid of mRFP-actin was a gift from A. Weaver (Vanderbilt University) and TOPflash reporter plasmid was from E. Lee (Vanderbilt University).

### Cell culture

A431 (human epidermoid squamous carcinoma cell line), HEK293 (a human embryonic kidney cell line), HCT-8, HCT-15, HCT-116, and HT-29 (human colon carcinoma cell lines) were obtained from American Type Culture Collection (Manassas, VA) and maintained in DMEM (Invitrogen, Carlsbad, CA) supplemented with 10% fetal bovine serum (FBS; Invitrogen) and 2 mM L-glutamine, and kept in constant culture in a humidified incubator with 5% CO_2_ at 37°C.

### Transfection

For the live cell imaging, A431 cells were co-transfected with β-catenin-ΔGSK-PA-GFP and mRFP-actin (3∶1) by using FuGENE® HD (Roche, Basel, Switzerland). The cDNA of β-catenin-ΔGSK was subcloned into PA-GFP expressing vector by using SacII and BamHI restriction enzyme sites.

### Immunofluorescence

Immunofluorescence labeling for confocal microscopy was performed as described previously with minor modifications [60]. Briefly, cells were serum-starved for 18 to 24 h, subsequently stimulated by 1 µM LPA and/or 40 mM lithium chloride (LiCl) for 1–3 h, fixed in 3.7% formaldehyde in PBS for 30 min, and permeabilized by 0.3% Triton X-100 in PBS for 5 min. After permeabilization, the sample was blocked with PBS containing 10% heat-inactivated goat serum and 1% bovine serum albumin for 45 min. After incubation with primary antibody (against ß-catenin, a-catenin, E-cadherin, PCNA, EEA1, Rab11, Golign97) in PBS containing 3% BSA for 1 h, cells were washed with PBS and incubated with secondary antibodies (Alexa-Fluor™488-anti-rabbit and/or Alexa-Fluor™568-anti-mouse IgG) for 45 min. Each sample was washed, mounted, and monitored with an LSM-510 laser scanning confocal microscope (Carl Zeiss, Germany; 40× EC Plan-NEOFLUAR; NA 1.3).

### Pulse-chase/ Live-cell imaging

Cells expressing β-catenin-ΔGSK-PA-GFP and mRFP-actin were starved for 18 h and placed on a Zeiss LSM-510 stage equipped with a humidified and heated chamber (37°C). The AJ and nuclear areas of transfected cells were identified based on the mRFP-actin image (AJ region was densely labeled with actin, while the nuclear region is almost absent of staining), and the junctional area alone was activated by 405-nm excitation light. The green fluorescence (488 nm excitation) of each area was monitored at various time points over the course of 25 min. The fluorescence intensity was measured and compared using a Zeiss LSM-510 microscope and image analysis software. Raw nuclear and AJ associated fluorescence values were converted to nuclear/AJ ratios to remove bias from variability in cell size/morphology and initial fluorescence intensities captured at 0 h. Ratios (300–1500 sec) were then normalized against corresponding measurements at 0 h, resulting in values presented as the fold-change of nuclear/AJ ratios over time.

### Cell fractionation and immunoblot

After LPA stimulation, cells were washed in ice-cold PBS and collected by centrifuge at 700×g. The nuclear fraction and cytoplasmic fractions were extracted by using NE-PER® Nuclear and Cytoplasmic Extraction Reagents (Pierce Biotechnology, Rockford, IL) according to the manufacturer's manual. Isolated fractions were separated on 8% SDS-polyacrylamide gel and analyzed by immunoblot using Odyssey Infrared Imaging System (LI-COR, Inc., Lincoln, NE).

### Transcriptional Reporter Gene Assay

TCF/LEF-dependent transcriptional activity was assessed by using TOPflash reporter plasmid. Briefly, the reporter gene was transfected into cells grown to semi-confluence in 12-well plates, and cultured for a further 6–8 h. After overnight starvation, cells were stimulated by PBS or LPA (5 µM) in the presence or absence of LiCl (20 mM) or Wnt3a-L-cell conditioned media (collected by centrifugation at 1500 g for 10 min to remove cell debris) for 18 h. Luciferase activity was measured by using Steady-Glo Luciferase Assay System (Promega, Madison, WI) according to the manufacturer's instructions.

In order to see the effect of β-catenin-GFP or β-catenin-ΔGSK-GFP, they were co-transfected into cells with TOPflash reporter gene and the luciferase activity was measured with or without LPA stimulation, as described above.

### Data analysis and statistics

Data analysis was performed using SPSS, version 16 (SPSS Inc., Chicago, IL). Differences between cell treatments were examined using two-sided Student's t-tests, and were considered significant when P<0.05.

## Supporting Information

Figure S1 LPA induces redistribution of E-cadherin, α-catenin and β-catenin. A431 cells were stimulated with 1 µM LPA for 3 h and stained with Alexa-FluorTM 568-phalloidin and Alexa-FluorTM 488 anti-mouse IgG to detect F-actin (red) and E-cadherin (green), respectively. α- and β-catenin were visualized the same way. Images (scale bar = 50 µm) were captured with a Zeiss Axioplan-2 fluorescence microscope. LPA-treated cells displayed disorganized β-catenin, α-catenin, and E-cadherin expression, as expected in disintegrating AJ.
(3.16 MB EPS)

Figure S2 β-catenin is targeted to the perinuclear endocytic recycling compartment. A431 cells were serum-starved, treated with 1 µM LPA or PBS for 1 h, immunostained for indicated antibodies, and imaged with a Zeiss LSM510 confocal microscope (scale bar = 50 µm). In LPA-treated cells, β-catenin (green) was not colocalized with lysosome (red, LAMP1) or the Golgi (red, Golgin-97), but partially colocalized with early endosomes (red, EEA1).
(6.50 MB EPS)

Figure S3 Ca++-deprivation mimics LPA effects. A431 cells were serum-starved for 18 h, treated with 1 µM LPA or PBS for 1 h in the presence or absence of 1.8 mM Ca++ in serum-free media, immunostained for β-catenin, and imaged with a Zeiss LSM510 confocal microscope (scale bar = 50 µm). In normal, higher Ca++ levels (1.8 mM), no nuclear β-catenin was observed with or without the Wnt activator LiCl; in contrast, in low Ca++ concentrations, upon dissociation of AJ, β-catenin is transported to a perinuclear region (presumably the ERC) and translocated to the nucleus in the presence of LiCl. Compared with LPA stimulation, side-by-side, results with low Ca++ were not as clearly interpretable, possibly due to disturbances of cell shape.
(1.97 MB EPS)

Figure S4 Proteasome inhibitor, MG132, promotes β-catenin translocation to nucleus. A431 cells were serum-starved, treated with 1 µM LPA or PBS for 1 h, subsequently treated with 40 mM LiCl or 10 µM MG132 (a proteasome inhibitor) for 1 h, immunostained for β-catenin for 45 min, and examined with a Zeiss LSM510 confocal microscope (scale bar = 50 µm). LPA increased the nuclear accumulation of β-catenin in cells co-stimulated by MG132.
(3.81 MB EPS)

Figure S5 TCF/LEF-dependent transcriptional activity TOPflash analysis of Wnt3a treated A431. In order to measure TCF/LEF-dependent transcriptional activity of the A431 cell line, cells were transfected with a TOPflash reporter plasmid. After overnight starvation, cells were stimulated by either PBS or LPA, in the presence or absence of Wnt3a-L-cell conditional medium (Wnt3a) for 18 h, and were assessed for luciferase activity using the Steady-Glo Luciferase assay system. Both PBS and LPA alone showed negligible levels of transcriptional activity (N = 3). In contrast, cells treated with Wnt3a showed significant increase in signal (N = 3; P = 0.011), which was significantly reduced by co-treatment with LPA (N = 3; P = 0.023).
(0.48 MB EPS)

Figure S6 TCF/LEF-dependent transcriptional activity TOPflash analysis of Î^2^-catenin mutated A431 cells. In order to see the effect of Î^2^-catenin-GFP or Î^2^-catenin-GSK-GFP, each of these constructs was co-transfected into A431 cells with TOPflash reporter gene. After overnight starvation, cells were stimulated by PBS or LPA, in the presence or absence of LiCl or Wnt3a for 18 h, and assessed for transcriptional activity using the Steady-Glo Luciferase assay system. Each PBS, Î^2^-catenin-GFP, and Î^2^-catenin-GFP+LPA alone showed negligible levels of transcriptional activity (N = 3). In contrast, Î^2^-catenin-GFP cells treated with LiCl showed a significant increase in signal (N = 3; P<0.0001). Further, Î^2^-catenin-GSK mutants exhibited significantly decreased signal upon LPA treatment (N = 3; P<0.0001). Oppositely, cells treated with Wnt3a significantly increased gene expression (N-3; P<0.0001).
(0.68 MB EPS)

Figure S7 Colon cancer cell lines display no discernable pattern of β-catenin transcriptional expression. In order to measure TCF/LEF-dependent transcriptional activity of colon cancer cells, HCT-8, HCT-15, HCT-116, and HT-29 cell lines were transfected with a TOPflash reporter plasmid. After overnight starvation, cells were stimulated by either PBS or LPA, in the presence or absence of LiCl for 18 h, and were assessed for luciferase activity using the Steady-Glo Luciferase assay system. Colon cancer cells exhibited no discernable pattern of expression.
(0.72 MB EPS)
